# Recent achievements in synthesis of anthracene scaffolds catalyzed transition metals

**DOI:** 10.3389/fchem.2025.1545252

**Published:** 2025-03-03

**Authors:** Fadhil Faez Sead, Vicky Jain, R. Roopashree, Aditya Kashyap, Suman Saini, Girish Chandra Sharma, Pushpa Negi Bhakuni, Mosstafa Kazemi, Ramin Javahershenas

**Affiliations:** ^1^ Department of Dentistry, College of Dentistry, The Islamic University, Najaf, Iraq; ^2^ Department of Medical Analysis, Medical Laboratory Technique College, The Islamic University of Al Diwaniyah, Al Diwaniyah, Iraq; ^3^ Department of Medical Analysis, Medical Laboratory Technique College, The Islamic University of Babylon, Babylon, Iraq; ^4^ Marwadi University Research Center, Department of Chemistry, Faculty of Science Marwadi University, Rajkot, Gujarat, India; ^5^ Department of Chemistry and Biochemistry, School of Sciences, JAIN (Deemed to be University), Bangalore, Karnataka, India; ^6^ Centre for Research Impact and Outcome, Chitkara University Institute of Engineering and Technology, Chitkara University, Rajpura, Punjab, India; ^7^ Department of Chemistry, Chandigarh Engineering College, Chandigarh Group of Colleges-Jhanjeri, Punjab, India; ^8^ Department of Applied Sciences-Chemistry, NIMS Institute of Engineering and Technology, NIMS University Rajasthan, Jaipur, India; ^9^ Department of Allied Science, Graphic Era Hill University, Bhimtal, Uttarakhand, India; ^10^ Graphic Era Deemed to be University, Dehradun, Uttarakhand, India; ^11^ Young Researchers and Elite Club, Islamic Azad University, Tehran, Alborz, Iran; ^12^ Department of Organic Chemistry, Faculty of Chemistry Urmia University, Urmia, Iran

**Keywords:** anthracene, transition metals (Cr and Fe), synthesis, nanocatalys, catalyst

## Abstract

In the last 10 years, the synthesis of anthracene scaffolds has attracted considerable interest because of their distinctive electronic characteristics and various uses in organic electronics, photovoltaics, and therapeutics. Anthracene, a polycyclic aromatic hydrocarbon, is valued for its lightweight, stability, and electron transport capabilities, making it a key building block in advanced materials. Traditional synthesis methods often face challenges such as low selectivity and harsh conditions. However, recent advancements in transition metal-catalyzed reactions have transformed the field, offering more efficient and versatile approaches. This review examines methodologies utilizing transition metal catalysts like palladium, zinc, indium, cobalt, gold, iridium, rhodium and ruthenium, which have enabled novel synthetic pathways and selective formation of substituted anthracenes through cross-coupling reactions. The function of ligands, including phosphines and N-heterocyclic carbenes, in improving reaction efficiency and selectivity is also examined. The shift towards greener methodologies is noted, with a focus on minimizing waste and reducing toxic reagents. The shift towards greener methodologies is noted, with a focus on minimizing waste and reducing toxic reagents. Several case studies demonstrate the successful application of these techniques, highlighting the structural diversity and functional potential of anthracene derivatives in various applications.

## 1 Introduction

The synthesis of anthracene frameworks has become a crucial field of study in organic chemistry, propelled by the compound’s distinctive electronic characteristics and its wide-ranging uses in areas such as organic electronics, photovoltaics, and medicinal chemistry. Anthracene, a polycyclic aromatic hydrocarbon (PAH), features a flat structure and excellent electron mobility, positioning it as a prime candidate for the innovation of advanced materials. The growing need for effective and adaptable synthetic methods has led chemists to investigate novel ways to create anthracene derivatives, especially using transition metal catalysis. Initially isolated in the early 1800s, anthracene has attracted considerable interest because of its wide-ranging uses in different domains, such as organic electronics, photovoltaics, and medicinal chemistry (Bass et al., 2024; Aydemir et al., 2023; Shi et al., 2022; Khandaka et al., 2023; Santra et al., 2012; Zhang et al., 2014; Modi et al., 2015).

Anthracene, a tricyclic aromatic hydrocarbon consisting of three fused benzene rings, has long been a subject of fascination for chemists and materials scientists alike. Its unique electronic properties, structural rigidity, and potential for functionalization have positioned anthracene as a crucial building block in various fields, including organic electronics, photovoltaics, and medicinal chemistry. The past decade has witnessed a surge in research focused on developing efficient and versatile methods for synthesizing anthracene scaffolds, driven by the compound’s diverse applications and the continuous demand for novel materials with enhanced properties (Kaewpuang et al., 2024; Modi et al., 2015; Climent et al., 2013; Climent et al., 2012).

Traditional synthetic methods for anthracene derivatives often face significant limitations, including low selectivity, harsh reaction conditions, and lengthy reaction times. These challenges have necessitated the development of more efficient strategies that can overcome these obstacles while maintaining high yields and selectivity. Recent advancements in transition metal-catalyzed reactions have revolutionized the field, providing chemists with powerful tools to construct anthracene frameworks with unprecedented efficiency and versatility.

Historically, traditional methods for synthesizing anthracene derivatives have been fraught with challenges, including low selectivity, harsh reaction conditions, and extended reaction times. These limitations have spurred the scientific community to explore more efficient and versatile synthetic strategies.

**Table:** 

Mosstafa Kazemi was born in Ilam, Iran. He has received MS degree in organic chemistry from Ilam University in 2013, his Ph.D. degree in organic chemistry from Ilam University in 2018. Dr. Kazemi is interested in the development of novel synthetic methods, nanocatalysts and particularly involving the application of Magnetic nanocatalysts in chemical reactions.	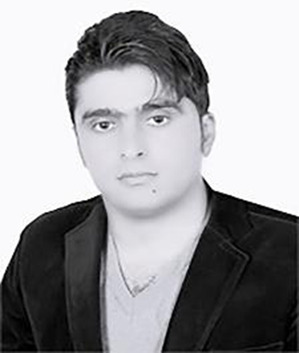
Ramin Javahershenas was born in Urmia, Iran, in 1971. He received his BSc in applied chemistry from Tabriz University, Tabriz, Iran in 1993, his MSc in organic chemistry from Urmia University, Urmia, Iran under the supervision of Professor Naser Ardabilchi in 1999, and his PhD in organic chemistry from Urmia University, Urmia, Iran under the supervision of Professor Jabbar Khalafy in 2017. His research interests canter around organic synthesis and include heterocyclic synthesis, asymmetric synthesis, natural products synthesis, synthetic methodology, and applications of various catalysts in multicomponent reactions.	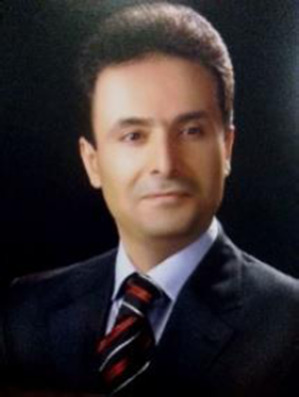

In recent years, transition metal-catalyzed reactions have revolutionized the synthesis of anthracene scaffolds, offering novel pathways that enhance both efficiency and selectivity. Traditionally, the synthesis of anthracene and its derivatives relied on classical organic reactions, such as Diels-Alder cycloadditions, Friedel-Crafts acylations, and oxidative photocyclization. While these methods have proven effective, they often suffer from limitations including low selectivity, harsh reaction conditions, and extended reaction times. Moreover, the increasing emphasis on sustainable chemistry has necessitated the development of more environmentally friendly synthetic approaches (Zhang et al., 2017; Anastas and Warner, 1998; Noyori, 2013; Anastas and Kirchhoff, 2002; Suzuki, 2011).

Transition metals, such as palladium, platinum, nickel, and copper, have been extensively utilized as catalysts in various cross-coupling reactions, including Suzuki-Miyaura, Sonogashira, and Negishi coupling. These methodologies have enabled the selective formation of substituted anthracenes, allowing for the introduction of diverse functional groups that enhance the properties of the resulting materials (Hartwig, 2008; Suzuki and Company, 2011; Jacobsen et al., 2010; Wencel-Delord et al., 2012).

The integration of these coupling strategies with other synthetic methodologies, such as cyclization and functionalization, has led to the discovery of previously inaccessible anthracene derivatives, expanding the scope of anthracene chemistry. This integration underscores the versatility of transition metal catalysis in expanding the structural diversity and functional capabilities of anthracene-based materials. The role of ligands in transition metal-catalyzed reactions cannot be overstated. Ligands play a crucial role in modulating the reactivity and selectivity of metal catalysts, influencing the overall efficiency of the synthetic process. Innovative ligand systems, including phosphines and N-heterocyclic carbenes (NHCs), have been developed to optimize catalytic performance, enabling the formation of complex anthracene structures under milder conditions. A critical aspect of these catalytic processes is the role of ligands, which are pivotal in enhancing reaction efficiency and selectivity (Hartwig, 2010; Beller and Bolm, 2004; Negishi, 2002).

Moreover, the environmental implications of these synthetic strategies cannot be overlooked. The shift towards greener methodologies that minimize waste and reduce reliance on toxic reagents is a significant trend in contemporary organic synthesis. This aligns with the broader goals of sustainable chemistry, aiming to reduce the environmental footprint of chemical processes while maintaining high efficiency and selectivity. Several case studies exemplify the successful application of transition metal catalysis in the synthesis of anthracene scaffolds. These examples not only highlight the structural diversity and functional prowess of the resulting anthracene derivatives but also demonstrate their potential in applications ranging from light-emitting diodes to sensor technologies (Crabtree, 2009; Colacot, 2015; Johansson Seechurn et al., 2012).

In recent years, the field of anthracene synthesis has undergone a paradigm shift with the advent of transition metal-catalyzed reactions. These methodologies have opened up new avenues for constructing complex anthracene scaffolds with unprecedented efficiency and selectivity. The use of transition metals as catalysts has not only expanded the synthetic toolkit available to chemists but has also enabled the creation of previously inaccessible anthracene derivatives. The use of transition metal catalysis in organic synthesis has transformed the field, providing effective methods for creating carbon-heteroatom bonds, and carbon-carbon (Tsuji, 2005; Trost and Crawley, 2003).

In the context of anthracene synthesis, transition metals such as platinum, nickel, palladium, and copper have emerged as particularly effective catalysts. These metals enable a variety of transformations, such as cross-coupling reactions, cyclizations, and C-H activations, which are essential for building the anthracene core and adding different functionalities. In addition to enhancing synthetic efficiency, the environmental implications of these methodologies have gained increasing attention. The shift towards greener synthetic strategies that minimize waste and reduce the reliance on toxic reagents is a critical consideration in contemporary organic synthesis. Recent advancements in transition metal-catalyzed reactions have demonstrated the potential for developing sustainable methodologies that align with the principles of green chemistry, thereby addressing the environmental challenges associated with traditional synthetic approaches (Molnár, 2013; van Leeuwen, 2004).

## 2 Chemical structure and properties

### 2.1 Chemistry

Anthracene is a polycyclic aromatic hydrocarbon (PAH) made up of three interconnected benzene rings set in a linear arrangement. Anthracene has a planar structure, its chemical formula is C_14_H_10_, which allows for effective π-π stacking interactions between molecules. This planarity contributes to its electronic properties and stability. The three benzene rings are fused together, sharing two carbon atoms at each junction. This leads to a continuous conjugated system that improves its capacity to conduct electricity and absorb light. Anthracene’s carbon atoms are sp^2^ hybridized, creating a network of double bonds (C=C) and single bonds (C-C). The resonance structure generated by the alternating double bonds enhances the molecule’s stability. Anthracene can undergo various chemical reactions, allowing for the introduction of substituents at different positions on the rings. This functionalization can modify its properties and expand its applications (Clar, 1964; Guo et al., 2018; Salam et al., 2014; Alonso et al., 2010).

The key properties of anthracene include:

• Planarity and Conjugation: The planar structure of anthracene allows for effective π-π stacking interactions, which are crucial for its performance in various applications. The extended conjugation across the three benzene rings results in a relatively low energy gap between the highest occupied molecular orbital (HOMO) and the lowest unoccupied molecular orbital (LUMO).
• Optical Properties: Anthracene shows significant absorption in the ultraviolet-visible (UV-Vis) spectrum, peaking at a wavelength of approximately 254 nm. Its high fluorescence quantum yield makes it a superb candidate for light-emitting applications.
• Thermal Stability: Anthracene is thermally stable up to approximately 300°C, which allows it to maintain its structural integrity under various processing conditions.
• Solubility: Anthracene dissolves in organic solvents like benzene, toluene, and chloroform, but its solubility in water is restricted. This property is essential for its operation in organic electronic devices.

The extended π-conjugation in anthracene leads to a relatively low energy gap between the HOMO and LUMO, facilitating efficient absorption and emission of light. This property is crucial for its applications in optoelectronic devices (Park et al., 2008).

### 2.2 Reactions pathways and parameters

Among the most widely employed strategies in transition metal-catalyzed anthracene synthesis are cross-coupling reactions. The Suzuki-Miyaura coupling, catalyzed primarily by palladium complexes, has proven exceptionally versatile in forming biaryl bonds, a key step in assembling the anthracene framework (Baviera and Donate, 2021; Robertson, 1930; Menendez et al., 2014).

Similarly, the Sonogashira coupling, which involves the palladium-catalyzed coupling of terminal alkynes with aryl or vinyl halides, has been instrumental in synthesizing alkynylated anthracene precursors. These intermediates can subsequently undergo cyclization to form the desired anthracene scaffolds (Oliveira et al., 2020; Zehm et al., 2008).

Another significant advancement in anthracene synthesis is the development of C-H activation methodologies. These approaches allow for the direct functionalization of C-H bonds, bypassing the need for pre-functionalized starting materials. Transition metals, particularly palladium and rhodium, have shown remarkable activity in catalyzing such transformations (Becker, 1993; Yoshizawa and Klosterman, 2014; Kastrati et al., 2023).

The success of transition metal-catalyzed reactions in anthracene synthesis is intrinsically linked to the design and selection of appropriate ligands. Ligands play a crucial role in modulating the reactivity and selectivity of the metal catalyst, often determining the outcome of the reaction. Recent years have seen significant advancements in ligand design, with a focus on developing systems that enhance catalytic performance while allowing for milder reaction conditions (Zarren et al., 2019; Floyd et al., 1976; Harvey, 2004).

Phosphine ligands have long been staples in transition metal catalysis, and their application in anthracene synthesis is no exception. The electronic and steric properties of phosphines can be finely tuned, allowing for optimization of catalyst activity and selectivity. For instance, the use of bulky, electron-rich phosphines such as SPhos and XPhos in Suzuki couplings has enabled the efficient synthesis of sterically hindered anthracene derivatives (Burrell et al., 2001; Han et al., 2023).

N-Heterocyclic carbenes (NHCs) have emerged as powerful alternatives to phosphine ligands in many catalytic systems. Their strong σ-donating properties and unique steric characteristics often result in catalysts with enhanced stability and activity. In the context of anthracene synthesis, NHC-ligated palladium complexes have shown exceptional performance in challenging cross-coupling reactions (Du C.-B. et al., 2024; Fan et al., 2024).

As the field of anthracene synthesis advances, there is an increasing emphasis on developing methodologies that align with the principles of green chemistry. This shift is driven by both environmental concerns and regulatory pressures, pushing researchers to design more sustainable synthetic routes (Bass et al., 2024; Anastas and Warner, 1998; Anastas and Kirchhoff, 2002; Anastas and Kirchhoff, 2002; Zehm et al., 2008).

One approach to enhancing the sustainability of transition metal-catalyzed anthracene synthesis is the development of recyclable catalyst systems. Heterogeneous catalysts, in particular, offer the advantage of easy separation and potential reuse (Zhao et al., 2023; Khan, 2024).

The exploration of alternative reaction media, such as water or ionic liquids, represents another avenue for making anthracene synthesis more environmentally friendly. These systems can potentially reduce the use of volatile organic solvents and improve reaction efficiency (Malik and Müller, 2016; Zhu and Zhu, 2013).

### 2.3 Applications and future directions

**FIGURE 1:**
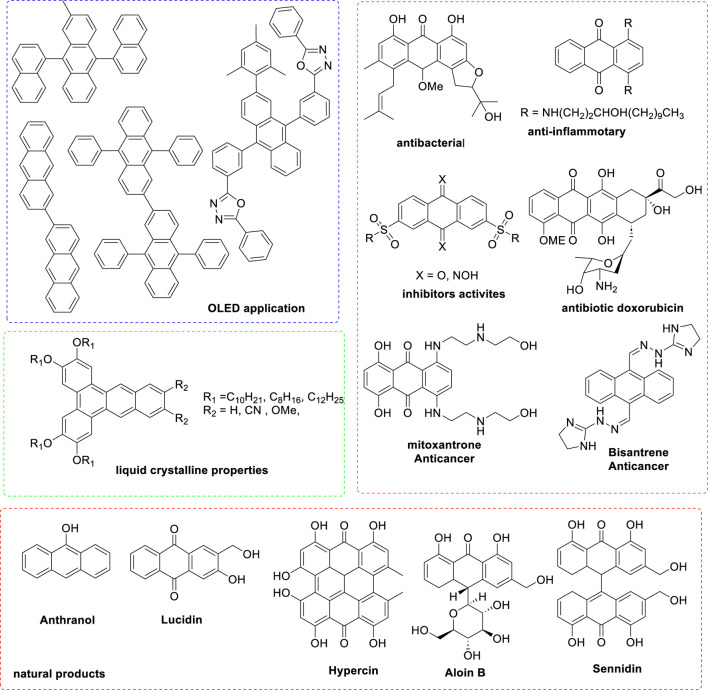
Some of anthracene derivatives.

The recent achievements in transition metal-catalyzed synthesis of anthracene scaffolds have not only expanded the synthetic toolkit but have also opened up new possibilities for applications. The ability to access structurally diverse and highly functionalized anthracenes has implications across various fields (Figure 1) (Wang et al., 2012; Vorona et al., 2019).

In the realm of organic electronics, anthracene derivatives synthesized through these novel methods have shown promise as materials for organic field-effect transistors (OFETs) and organic light-emitting diodes (OLEDs). The work of Wang *et al.* demonstrates how tailored anthracene scaffolds, synthesized via palladium-catalysed methods, can be utilized to create high-performance blue OLEDs (Zhang et al., 2019; Khan et al., 2023).

Anthracene’s planar structure allows for effective π-π stacking interactions, which are crucial for its performance in electronic applications. The compound exhibits strong absorption in the ultraviolet-visible (UV-Vis) region, making it an excellent candidate for light-absorbing materials. Its high electron mobility and stability further enhance its utility in organic semiconductor devices. In OLEDs, anthracene derivatives are often used as emissive materials due to their ability to emit light efficiently when excited by an electric current. The incorporation of anthracene into OLEDs has led to the development of devices with improved brightness and colour purity, making them suitable for displays and lighting applications (Becker, 1993; Ihmels et al., 2000; Abou-Hatab et al., 2017).

In OFETs, anthracene serves as a semiconductor material, where its high charge carrier mobility contributes to enhanced device performance. The ability to tune the electronic properties of anthracene through chemical modifications allows for the design of materials that meet specific performance criteria, further driving advancements in organic electronics (Pozzo et al., 2001; Huang et al., 2012).

Anthracene’s unique properties have led to its widespread use in various fields, including:

#### 2.3.1 Organic electronics

In organic electronics, anthracene is valued for its high charge carrier stability and mobility. These properties make it an ideal candidate for use in (Chen et al., 2018; Van Damme and Du Prez, 2018; Fang et al., 2017):

• Organic Light-Emitting Diodes (OLEDs): Anthracene derivatives are employed as emissive materials due to their ability to emit light efficiently when excited by an electric current. The incorporation of anthracene in OLEDs enhances brightness and colour purity, making them suitable for lighting and display applications.
• Organic Field-Effect Transistors (OFETs): Anthracene serves as a semiconductor material, where its high charge carrier mobility contributes to improved device performance. The ability to chemically modify anthracene allows for the design of materials that meet specific performance criteria.

#### 2.3.2 Photonics

Anthracene’s strong absorption in the ultraviolet-visible (UV-Vis) region and its ability to emit light make it a valuable material in photonics (Kivrak et al., 2017; Zhao et al., 2017; Khazaei et al., 2015). Applications include:

• Light-Emitting Materials: Anthracene derivatives are used in the development of materials that emit light in various colours, essential for creating efficient and tuneable light sources.
• Sensors: The photophysical properties of anthracene enable its use in sensors for detecting environmental pollutants and biological molecules. The fluorescence of anthracene can be quenched or enhanced in the presence of specific analytes, allowing for sensitive detection.

The pharmaceutical industry has also benefited from these advancements. The ability to rapidly generate libraries of functionalized anthracenes has accelerated drug discovery efforts. In medicinal chemistry, anthracene and its derivatives have shown significant potential as therapeutic agents: (Brabec et al., 2011; Hurley, 2002; Ho et al., 2007; Wu et al., 2014; Okumoto et al., 2006; Ito et al., 2003; Kouam et al., 2007; Corrêa et al., 2013; Soldi et al., 2015; Lown, 1988; Venitt et al., 1998; Iyengar et al., 1997).

• Anticancer Agents: Certain anthracene derivatives have demonstrated the ability to intercalate with DNA, disrupting cellular processes and exhibiting anticancer activity. Research has focused on synthesizing novel anthracene-based compounds with enhanced efficacy against various cancer types.
• Antimicrobial and Anti-inflammatory Properties: Anthracene derivatives have also been investigated for their antimicrobial and anti-inflammatory activities. These properties make them promising candidates for developing new drugs to combat infections and inflammatory diseases.
• Drug Delivery Systems: The unique properties of anthracene allow for its incorporation into drug delivery systems, where it can enhance the solubility and bioavailability of therapeutic agents.

Anthracene’s unique properties and versatility have established it as a critical compound in various scientific and industrial fields. Its role in organic electronics, photonics, and medicinal chemistry underscores its importance and potential for future innovations. Continued research and development of anthracene derivatives are expected to further expand its applications and enhance its contributions to technology and healthcare.

## 3 Transition metal catalysis

Over the last few decades, transition metal catalysis has transformed organic synthesis, allowing for the synthesis of new and efficient methods to build intricate molecular structures. The unique electronic properties and diverse coordination geometries of transition metals make them exceptionally versatile catalysts, capable of mediating a wide range of chemical transformations with high selectivity and efficiency. Transition metals, particularly those in the d-block of the periodic table, possess partially filled d-orbitals that allow them to form stable complexes with organic molecules. This ability to coordinate with various ligands and substrates is fundamental to their catalytic activity. Chemists can adjust the oxidation state and coordination environment of the metal centre to fine-tune the reactivity and selectivity of these catalysts, frequently accomplishing transformations that would be difficult or unfeasible with conventional organic reagents (Heravi et al., 2021; Dandia et al., 2015; Inamdar et al., 2013; Khatun et al., 2014; Iordanidou et al., 2018; Saha et al., 2009; Hussain et al., 2017; Kumar et al., 2014).

The significance of transition metal catalysis in organic synthesis is immense. From the advancement of palladium-catalyzed cross-coupling reactions, awarded the 2010 Nobel Prize in Chemistry, to the latest progress in gold catalysis and photoredox catalysis, transition metals persist in propelling innovation in synthetic methods. As research in this field progresses, new catalysts and reactions are constantly being discovered, further expanding the boundaries of what is possible in organic synthesis. Transition metals have emerged as pivotal catalysts in organic synthesis, revolutionizing the way complex molecules are constructed. These metals, including palladium, platinum, nickel, and copper, possess unique electronic properties that enable them to facilitate a wide range of chemical transformations. Transition metals can assume different oxidation states and create stable complexes with a variety of ligands, which increases their reactivity and selectivity in catalytic reactions (Parte et al., 2024; Albaladejo et al., 2013; Sadeghzadeh, 2016; Baghbanian, 2014; Kidwai et al., 2012; Reddy and Jeong, 2016; Balwe et al., 2017; Khafaei et al., 2021; Lak et al., 2021; Sabernezhad, 2021; Du Y. et al., 2024).

The use of transition metals in catalysis offers several advantages over traditional methods. Firstly, transition metal catalysts often exhibit high activity, enabling reactions to proceed under milder conditions and with shorter reaction times. This efficiency not only reduces energy consumption but also minimizes the formation of by-products, leading to cleaner reactions. For example, palladium-catalysed cross-coupling reactions, including the Suzuki and Heck reactions, have become established techniques for creating carbon-carbon bonds in organic synthesis, enabling the swift construction of intricate molecular structures (Farokhian et al., 2021; Bakhshi et al., 2021; Amjadian et al., 2021; Kahrizi et al., 2021; Rabbani and Safdary, 2021; Sead et al., 2025; Asadi and Jalilian, 2021; Mahboub Khomami et al., 2021; Zeidali et al., 2021).

Secondly, transition metal catalysts can provide exceptional selectivity, enabling the formation of specific products in the presence of multiple functional groups. This selectivity is particularly valuable in the synthesis of pharmaceuticals and agrochemicals, where the precise control of stereochemistry and functionalization is crucial. The development of chiral transition metal catalysts has further expanded the scope of asymmetric synthesis, allowing for the production of enantiomerically pure compounds (Jalali Sarvestani and Charehjou, 2021; Saffariha et al., 2021; Hassanpour, 2021; Jalilian, 2020).

Moreover, the versatility of transition metal catalysts extends to their ability to catalyse a diverse array of reactions, including oxidation, reduction, and C–H activation. This broad applicability makes them indispensable tools in modern organic synthesis. As research continues to advance, the design and optimization of transition metal catalysts are expected to yield even more efficient and sustainable synthetic methodologies (Rasouli et al., 2020; Ali et al., 2020a; Ali et al., 2020b; Bhattacharjee et al., 2018; Noyori, 2013).

### 3.1 Challenges and limitations

Despite the challenges, significant progress has been made in the synthesis of anthracene scaffolds using transition metal catalysis. While transition metal catalysis has significantly advanced the field of organic synthesis, several limitations and challenges of Existing Methodologies persist (Sead, 2025; El-Remailya and Hamad, 2015; Nguyen et al., 2019; Naeimi and Didar, 2017; Bullock, 2013).

These limitations and challenges can be classified into six primary categories: (1) the development of new catalysts; (2) environmental and safety concerns; (3) reactivity and selectivity issues; (4) ligand design and optimization; (5) metal residue and purity; and (6) cost and availability.

### 3.2 Future directions

The synthesis of anthracene scaffolds catalyzed by transition metals continues to evolve, driven by the need for more efficient, selective, and sustainable methodologies. While challenges remain, recent achievements in catalyst development, C–H activation, and cross-coupling reactions have expanded the synthetic toolbox available to chemists. Future research will likely focus on further optimizing these processes, exploring new catalytic systems, and integrating emerging technologies to address the limitations of existing methodologies. Although, the use of transition metals as catalysts in organic synthesis offers numerous advantages that have transformed the landscape of chemical manufacturing. Their catalytic efficiency, selectivity, versatility, and sustainability make them indispensable tools for chemists seeking to develop innovative and efficient synthetic methodologies. As research in this field continues to advance, the discovery of new transition metal catalysts and the optimization of existing ones will undoubtedly lead to further breakthroughs in organic synthesis (Trost, 1991; Bauer and Knölker, 2015; Prier et al., 2013; Sheldon et al., 2007; Ren et al., 2009).

## 4 Metal catalyzed synthesis of anthracene derivatives

In recent times, many publications have reported in the literature for the synthesis of anthracene derivatives based on using metal complexes as the catalyst. The present review focuses on the recent developments in the metal catalyzed syntheses of anthracene scaffolds.

### 4.1 Palladium catalyzed synthesis of anthracenes

**SCHEME 1:**
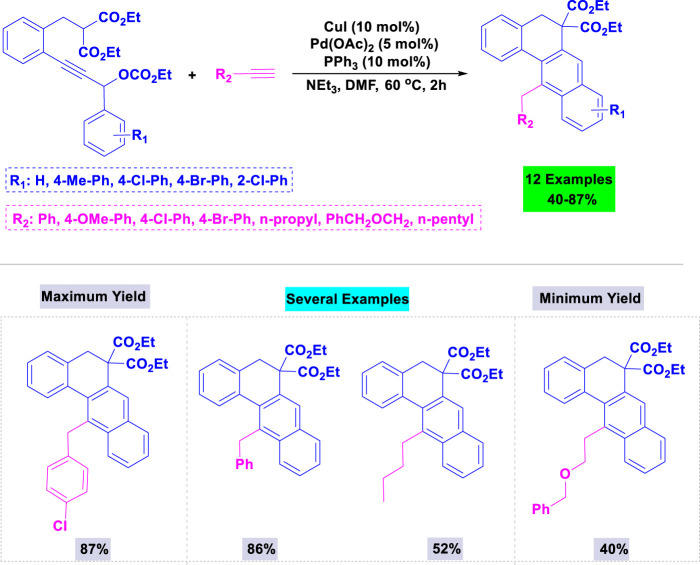
Synthesis of tetracyclic benz [a] anthracenes through palladium-catalyzed tandem C-H activation/biscyclization reaction of propargylic carbonates with terminal alkynes.

In 2009, Ren and colleagues reported that the combination of Pd(OAc)_2_ and PPh_3_ forms an efficient catalytic system for synthesizing tetracyclic benz [a]anthracene frameworks (Chan et al., 2009). The reaction involving propargylic carbonate and phenylacetylene was carried out with various catalysts and bases in different environments; the reaction did not succeed without the palladium catalyst. Scheme 1 illustrates the details of the palladium-catalyzed tandem C-H activation/biscyclization reaction involving propargylic carbonates and terminal alkynes.

**SCHEME 2:**
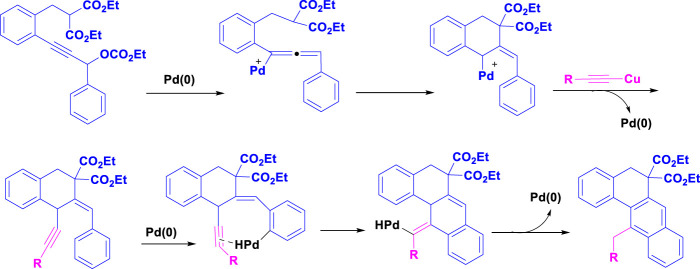
The plausible mechanism of Synthesis of tetracyclic benz [a]anthracenes through palladium-catalyzed tandem C-H activation/biscyclization reaction of propargylic carbonates with terminal alkynes.

Scheme 2 depicts a palladium-catalyzed tandem C-H activation/biscyclization reaction of propargylic carbonates with terminal alkynes to synthesize tetracyclic benz[a]anthracenes. The reaction begins with the oxidative addition of a Pd(0) catalyst to a C-H bond of the propargylic carbonate. This forms a Pd(II) intermediate. The Pd(II) intermediate undergoes carbonylation, inserting a CO molecule into the Pd-C bond. The resulting acyl-Pd(II) species then undergoes migratory insertion, inserting an alkene from the terminal alkyne into the Pd-C bond. Finally, reductive elimination occurs, releasing the tetracyclic benz[a]anthracene product and regenerating the Pd (0) catalyst for the next cycle.

The use of a palladium catalyst is crucial for the C-H activation and subsequent transformations. The propargylic carbonate and terminal alkyne are the key starting materials.

**SCHEME 3:**
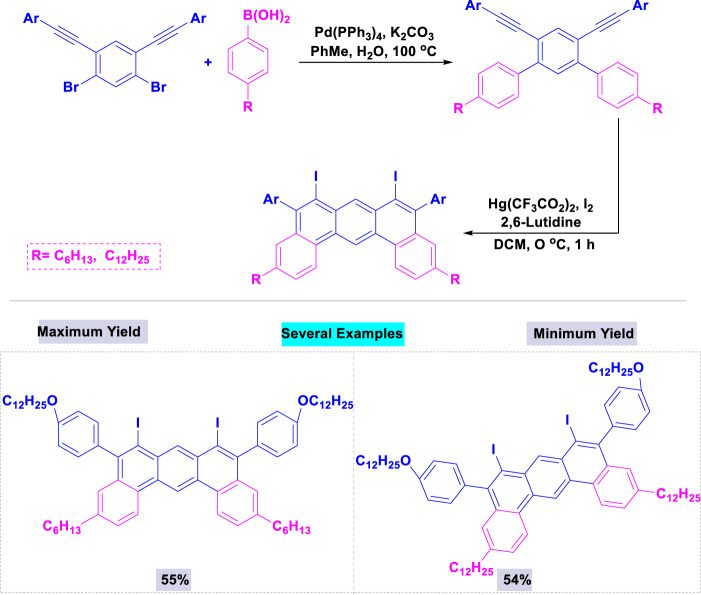
Synthesis of 6,8-diiododi-benzo [a,j]anthracenes through a multistep synthetic approach in the presence of catalytic amount of palladium.

A collection of fluorescent macrocycles constructed from 1,3-butadiyne-bridged dibenz[*a,j*]anthracene units has been synthesized with high yields via a multistep synthetic method using a catalytic amount of palladium (Umeda et al., 2012). The highest yield was observed with Pd(PPh)_3_ in combination with potassium carbonate, among the tested palladium and bases. Scheme 3 illustrates that the corresponding 6,8-diiododi-benzo[a,j]anthracenes were synthesized with good yields through double iodonium-induced electrophilic cyclization.

**SCHEME 4:**
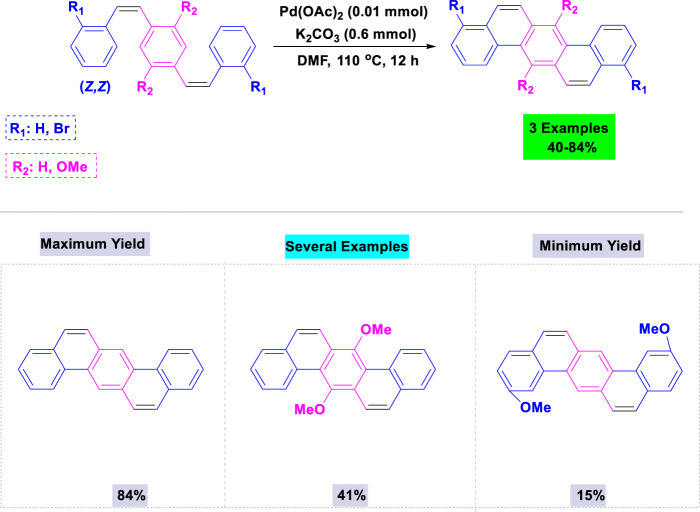
Synthesis of dibenz [*a,h*]anthracenes through Pd-catalyzed intramolecular double-cyclization of the corresponding (Z, Z)-p-styrylstilbene derivatives.

In 2012, Nishiyama and colleagues synthesized a wide variety of biologically active dibenz[a,h]anthracenes via Pd-catalyzed intramolecular double-cyclization of the respective (Z,Z)-p-styrylstilbene derivatives, which were easily prepared using the Wittig reaction (Kim et al., 2016). A variety of palladium catalysts and bases were tested in various solvents to identify the optimal conditions. The intramolecular double-cyclization of the relevant (Z,Z)-p-styrylstilbene derivatives was performed in a single pot with a catalytic quantity of Pd(OAc)_2_ and potassium carbonate in DMF under heating conditions (Scheme 4).

**SCHEME 5:**
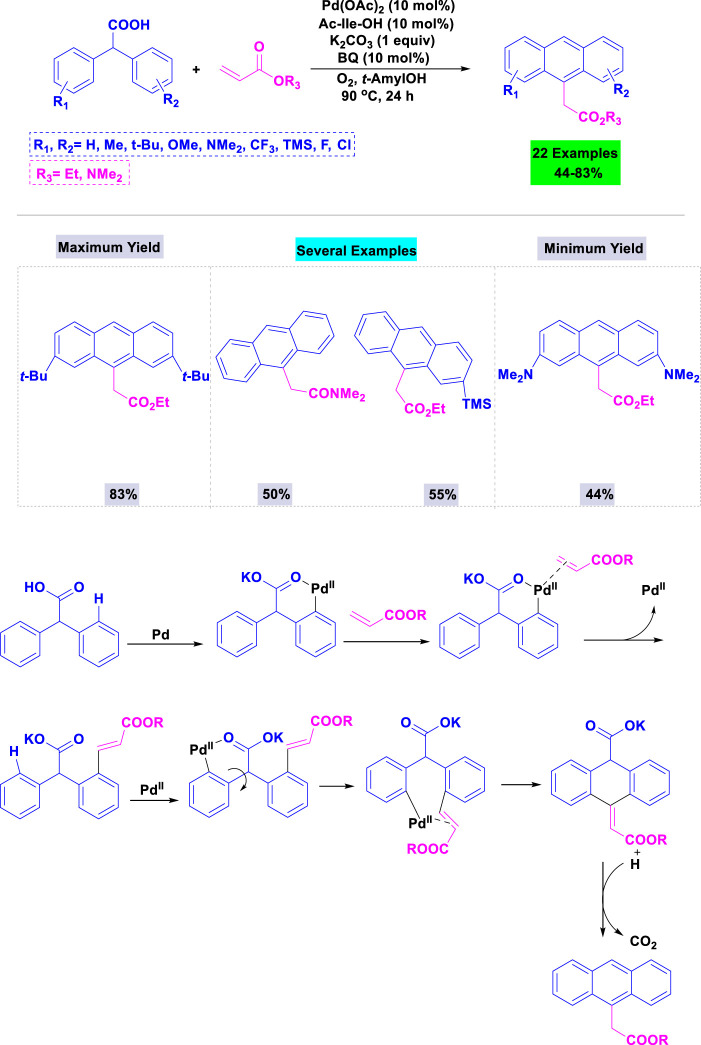
Synthesis and a plausible mechanism of synthesis of substituted anthracene derivatives through Pd(II)-catalyzed sp^3^ C–H alkenylation of diphenyl carboxylic acids with acrylates.

In 2016, Hong and colleagues reported the synthesis of substituted anthracene derivatives via palladium(II)-catalyzed tandem transformation using carboxylic acids as traceless directing groups (Psutka et al., 2015). A variety of parameters (bases, solvents and additives) were analyzed in the template condensation of diphenyl carboxylic acid with ethyl acrylate, catalyzed by Pd(OAc)_2_ with an amino acid-derived ligand; the highest yield was observed using potassium carbonate in t-amyl OH. Under standardized conditions, substrates featuring both electron-donating (Me-, t-Bu-, MeO-, and Me_2_N-) and electron-withdrawing groups (F-, Cl-, and CF_3_-) on the aryl moieties were successfully involved in these reactions, resulting in the desired anthracene products with moderate to good yields (Scheme 5).

This mechanism describes the synthesis of substituted anthracene derivatives using a palladium(II) catalyst (Scheme 5). It starts with the oxidative addition of Pd(II) to a diphenyl carboxylic acid. An acrylate then inserts into the Pd-C bond. A key step involves a C-H activation and cyclization to form the anthracene core. Finally, reductive elimination regenerates the Pd(II) catalyst and releases the substituted anthracene product, along with CO_2_.

**SCHEME 6:**
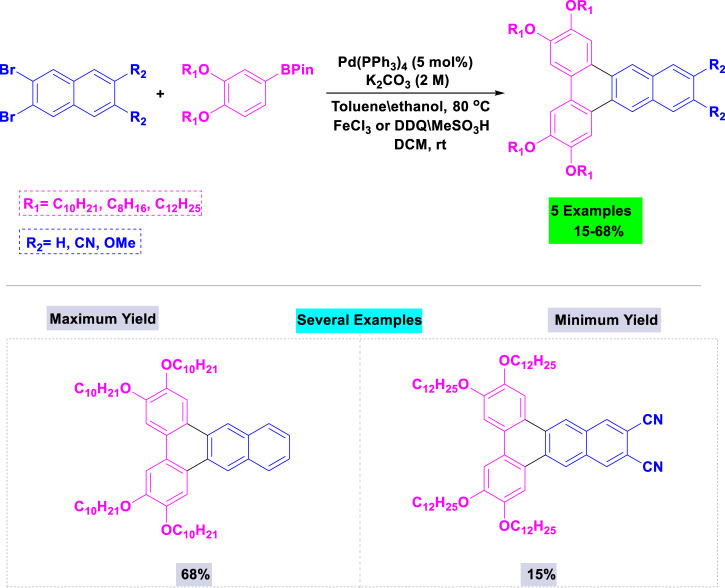
Synthesis of 2,3,5,6-tetraalkoxydi-benz [*a,c*]anthracenes through Pd(PPh_3_)_4_ catalyzed Suzuki coupling of the appropriate dibromonaphthalene and boronate ester, followed by an oxidative cyclization.

A broad range of 2,3,5,6-tetraalkoxydi-benz [a,c]anthracenes bearing substituents (H, OCH_3_, or CN) in the 11- and 12-positions were successfully synthesize by Maly and coworkers with good yields through Suzuki coupling of the appropriate dibromonaphthalene and boronate ester, followed by an oxidative cyclization in the presence of palladium (Park et al., 2018). These reactions were accomplished using 5 mol% of Pd(PPh_3_)_4_ and potassium carbonate in toluene/ethanol under thermal conditions (Scheme 6).

**SCHEME 7:**
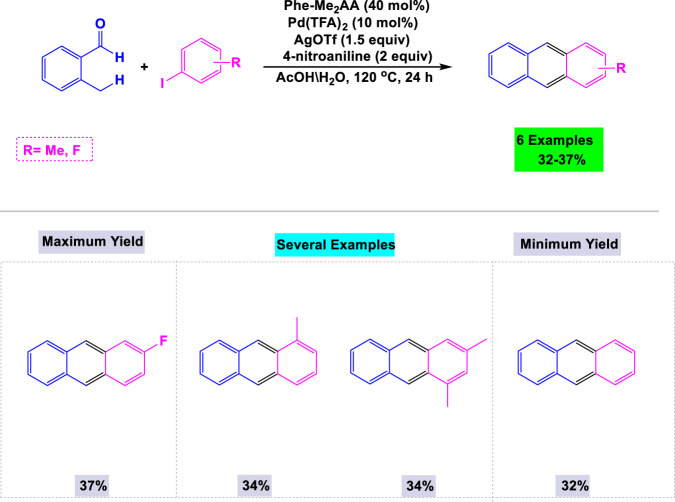
Synthesis of substituted anthracenes through Pd(I I)-Catalyzed sp^3^ C-H arylation and electrophilic aromatic cyclization.

In 2018, Park and colleagues reported that palladium catalyzes the reactions between o-tolualdehydes and aryl iodides to synthesize substituted anthracenes (Suchand and Satyanarayana, 2019). In their standardization experiments, the authors found that steric and electronic effects significantly influence the cyclization process leading to the formation of anthracenes. The details of the synthesis of substituted anthracenes from o-tolualdehydes and aryl iodides, utilizing Pd(II)-catalyzed sp³ C-H arylation and electrophilic aromatic cyclization, are illustrated in Scheme 7.

**SCHEME 8:**
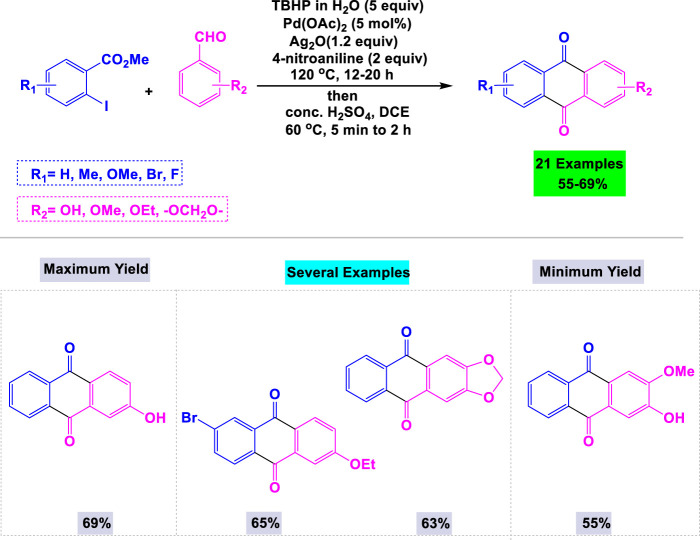
Synthesis of anthraquinones through [Pd]-catalyzed intermolecular direct acylation reaction.

A library of biologically active anthraquinones was synthesized in good yields via a [Pd]-catalyzed intermolecular direct acylation reaction. The subsequent acylation was accomplished through intramolecular Friedel–Crafts acylation (Kodomari et al., 2008). In this methodology, Satyanarayana and Such utilized 5 mol% of Pd(OAc)_2_ in the presence of Ag_2_O/TBHP to explore the reaction scope between methyl 2-iodobenzoate and various benzaldehydes in water under reflux conditions (Scheme 8). Notably, benchtop aldehydes were employed as non-toxic acylation agents in the critical [Pd]-catalyzed acylation process.

### 4.2 Zinc catalyzed synthesis of anthracenes

**SCHEME 9:**
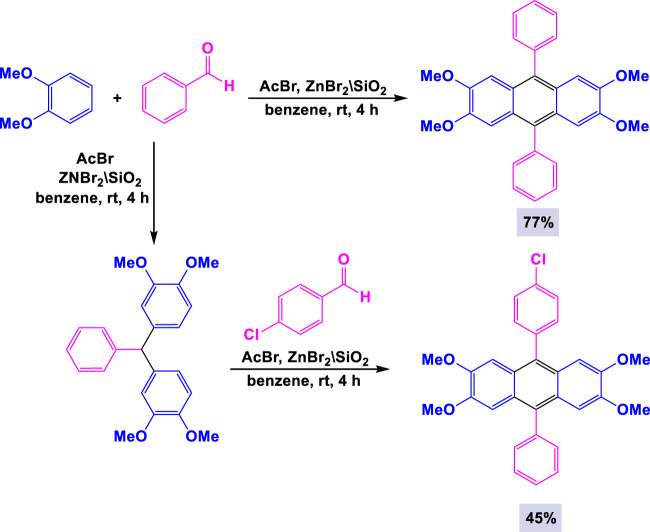
Synthesis of 9,10-diarylanthracene derivatives through ZnBr_2_/SiO_2_ catalyzed reaction of electron-rich arenes with aromatic aldehydes and acetyl bromide.

In 2008, Kodomari and colleagues employed silica gel-supported zinc bromide as a catalyst for the synthesis of 9,10-diarylanthracene derivatives. This synthesis was accomplished through the reaction of electron-rich arenes with acetyl bromide and aldehydes under mild conditions (Bhowmik et al., 2009). The experimental studies demonstrated that the yield of diarylanthracene derivatives was influenced by the ratio of arene to aldehyde. As illustrated in Scheme 9, the reaction of electron-rich arenes with aromatic aldehydes and acetyl bromide was catalyzed by ZnBr_2_/SiO_2_ in benzene under mild conditions, leading to favorable yields of the corresponding products.

**SCHEME 10:**
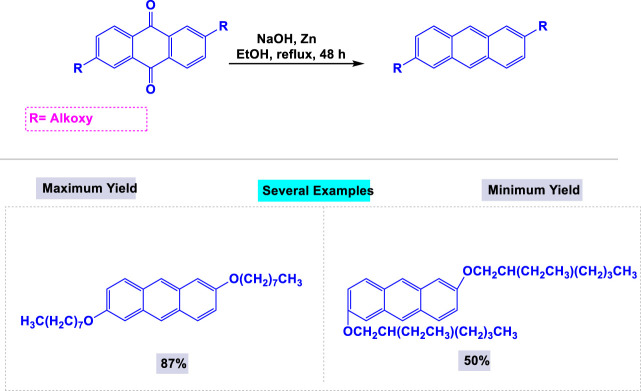
Synthesis of 9,10-diacetoxy-anthracenes through from anthraquinone and its derivatives via a single step reaction by using reductive Zn-pyridine system.

One year later, Bhowmik and coworkers reported a facile and effective synthetic methodology for preparing 9,10-diacetoxy-anthracene derivatives from anthraquinone and its derivatives through a one-step reaction using a reductive Zn-pyridine system in ethanol under reflux conditions (Scheme 10) (Pünner et al., 2013).

**SCHEME 11:**
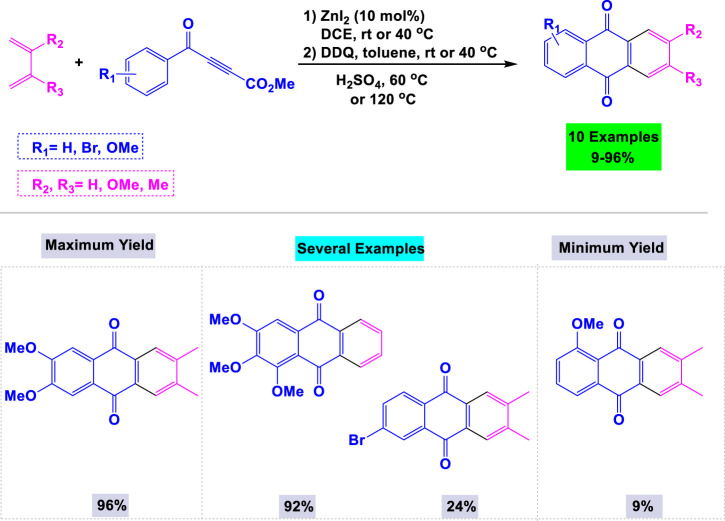
Synthesis of anthraquinone derivatives through Zinc iodide-catalyzed Diels–Alder reactions with 1,3-dienes and aroylpropiolates followed by intramolecular Friedel-Crafts cyclization.

Hilt and colleagues have developed an efficient synthetic route for the preparation of a wide range of symmetric and asymmetric anthraquinone derivatives, achieving good yields through zinc iodide-catalyzed Diels–Alder reactions with 1,3-dienes and aroylpropiolates, followed by intramolecular Friedel-Crafts cyclization (Agarwal et al., 2015). The scope and some limitations of these zinc iodide-catalyzed cyclization reactions are outlined in Scheme 11. The presence of more electron-donating alkyl or methoxy groups in the ring enhances the efficiency of the Friedel-Crafts cyclization.

**SCHEME 12:**
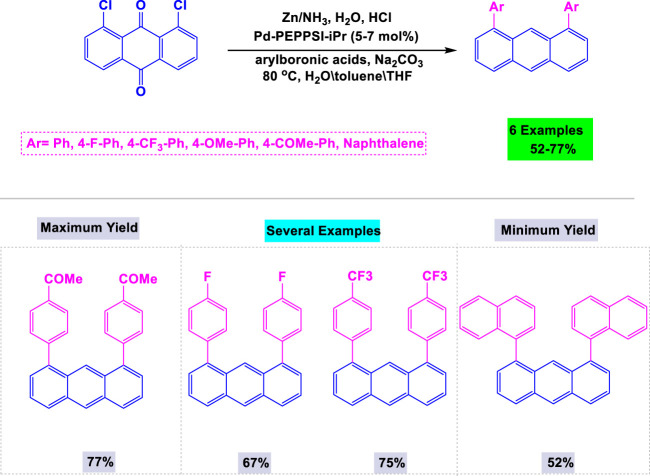
Synthesis of 1,8 -diaryl-anthracene derivatives from anthroquinones in the presence of zinc as the catalyst.

In 2015, Agarwal and colleagues introduced a novel and efficient method for synthesizing biologically active 1,8-diaryl-anthracene derivatives using zinc as a catalyst (Sivasakthikumaran et al., 2015). As illustrated in Scheme 12, the method involves the reduction of 1,8-dichloroanthraquinone, followed by aryl-aryl coupling under modified Suzuki-Miyaura reaction conditions, yielding good results for 1,8-diaryl anthracene derivatives.

**SCHEME 13:**
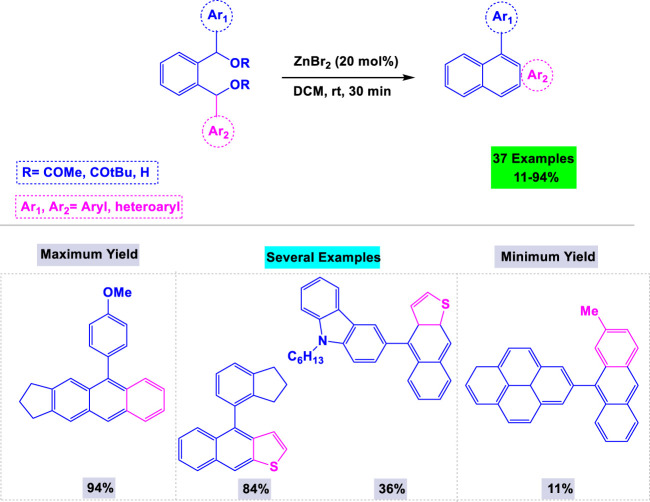
Synthesis of anthracenes through zinc bromide catalyzed one-pot regioselective annulation of unsymmetrical 1,2-phenylenebis (diaryl/diheteroarylmethanol).

In a separate publication on the synthesis of anthracenes, Mohanakrishnan and colleagues introduced zinc bromide as an efficient catalyst for the one-pot regioselective annulation of unsymmetrical 1,2-phenylenebis (diaryl/diheteroarylmethanol) (Imeni et al., 2023). They investigated the influence of the catalyst and solvent to optimize the reaction conditions, ultimately determining that 20 mol% of ZnBr in dichloromethane at ambient temperature was ideal for synthesizing anthracenes (Scheme 13). This system also demonstrated high activity in the preparation of other polycyclic aromatic hydrocarbons, such as tetracenes and naphtho [b]thiophenes.

### 4.3 Indium catalyzed synthesis of anthracenes

Multicomponent reactions (MCRs) are valuable tools in organic synthesis, facilitating the rapid construction of complex molecular structures from three or more reactants in a single reaction step. They are highly regarded for their efficiency, atom economy, and ability to introduce molecular diversity, rendering them essential in the development of pharmaceuticals and organic materials (Javahershenas and Nikzat, 2023; Javahershenas et al., 2024a; Javahershenas et al., 2024b; Javahershenas et al., 2024c; Nandi et al., 2009).

**SCHEME 14:**
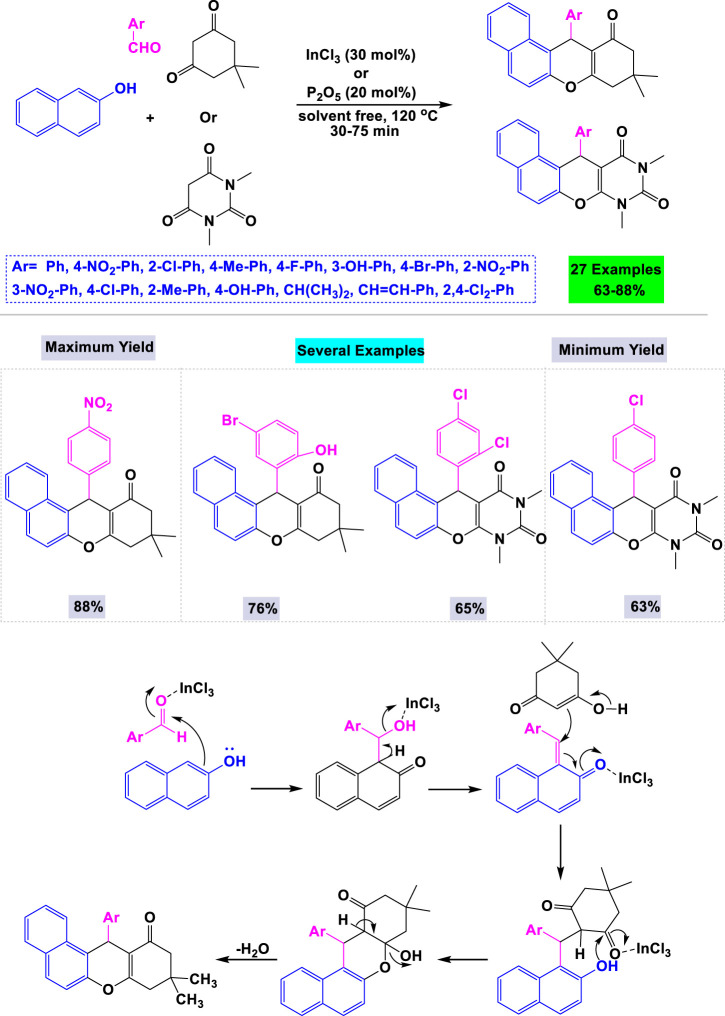
Synthesis and mechanistic rationale pathway of anthraquinone derivatives through cyclocondensation of aldehydes, *β*-naphthol and cyclic 1,3-di-carbonyl compounds catalyzed by InCl_3_.

**SCHEME 15:**
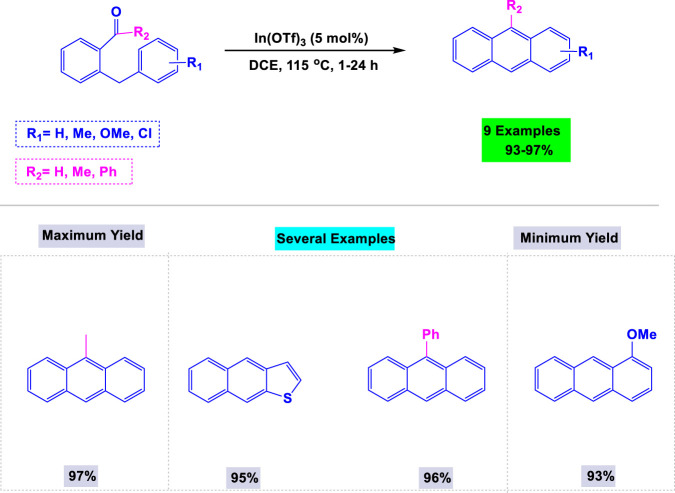
Synthesis of anthracene derivatives from 2-benzylic- or 2-allylbenzaldehydes using a catalytic amount of In(III) or Re(I) complexes.

In 2009, Nandi and colleagues developed a general and convenient synthetic method for preparing tetrahydrobenzo[a]xanthene-11-one and diazabenzo[a]anthracene-9,11-dione derivatives. This was achieved through a one-pot three-component cyclocondensation of aldehydes, β-naphthol, and cyclic 1,3-dicarbonyl compounds, catalyzed by InCl3 under solvent-free conditions (Scheme 15). (Kuninobu et al., 2011) The advantages of this catalytic system include appropriate reaction times, higher yields, mild reaction conditions, straightforward purification, and cost-effectiveness. A mechanistic pathway for the one-pot three-component cyclocondensation involving aldehydes, β-naphthol, and cyclic 1,3-dicarbonyl compounds catalyzed by InCl_3_ is illustrated in Scheme 14.

In 2011, Takai and colleagues developed a general and efficient synthetic methodology for the preparation of anthracene derivatives from 2-benzylic or 2-allylbenzaldehydes, utilizing a catalytic amount of In(III) or Re(I) complexes (Hueso-Falcón et al., 2014). Two key factors in optimizing the synthesis of anthracene derivatives were the concentration of the catalyst and the nature of the solvent. Details of these reactions are presented in Scheme 15. This methodology also demonstrated high efficiency in synthesizing other polycyclic aromatic hydrocarbons, including derivatives of naphthalene and naphtha [2,3-*b*]thiophene.

**SCHEME 16:**
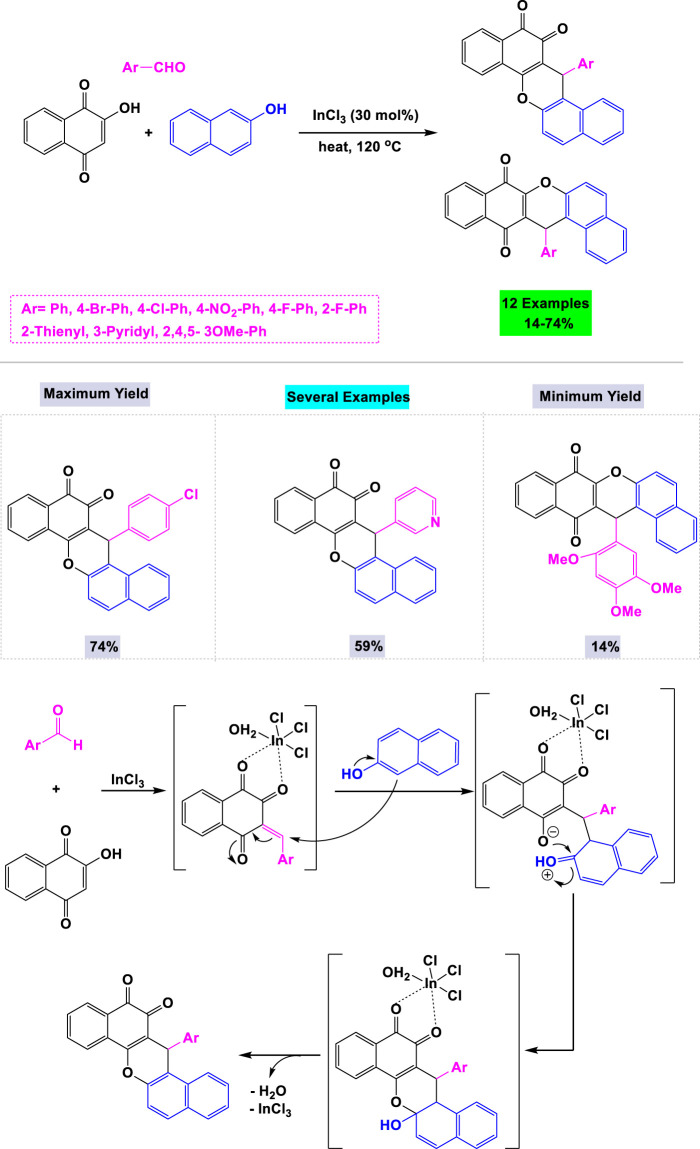
Synthesis and mechanistic rationale pathway of cytotoxic dibenzo [*a,h*]anthracenes through InCl3 catalyzed one-pot three-component reaction with 2-hydroxy-1,4-naphthoquinone, aromatic aldehydes, and 2-naphthol.

Estévez-Braun and colleagues reported the preparation of a library of cytotoxic dibenzo [*a,h*]anthracenes via an InCl_3_-catalyzed one-pot three-component reaction. This reaction involved 2-hydroxy-1,4-naphthoquinone, aromatic aldehydes, and 2-naphthol as synthetic inputs, all conducted under solvent-free conditions (Scheme 16). (Javahershenas et al., 2024d) To optimize the synthesis of cytotoxic dibenzo [a,h]anthracenes, various metal catalysts and solvents were evaluated. The highest yield was achieved using 30 mol% of InCl3 without solvent under thermal conditions. A mechanistic pathway for the InCl_3_-catalyzed one-pot three-component reaction is depicted in Scheme 16.

### 4.4 Cobalt catalyzed synthesis of anthracenes

**SCHEME 17:**
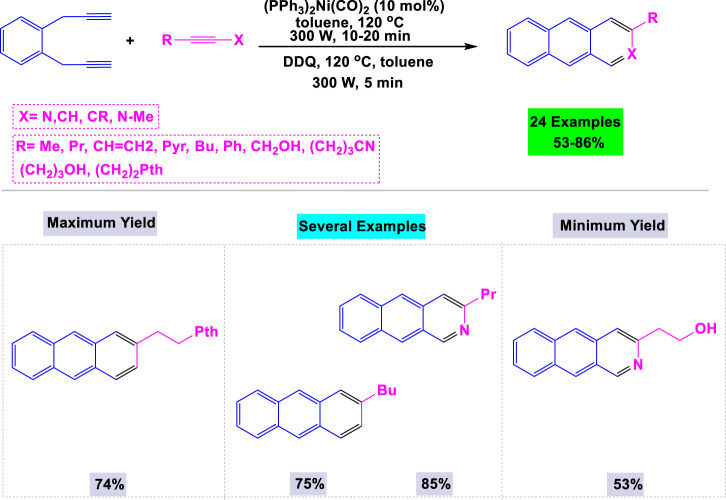
Synthesis of substituted anthracenes and azaanthracenes through Nickel/cobalt catalyzed [2 + 2 + 2] cyclotrimerization reactions.

Microwave-assisted synthesis is a contemporary organic technique that utilizes microwave radiation to heat reaction mixtures, resulting in significantly reduced reaction times and often enhanced product yields. This method is recognized for its efficiency, energy conservation, and capability to facilitate reactions that may be difficult to achieve under conventional thermal conditions. Zou et al. (2008) developed a general and effective microwave-assisted methodology for synthesizing substituted anthracenes and azaanthracenes in high yields through [2 + 2 + 2] cyclotrimerization reactions employing nickel and cobalt catalysts. The presence of a catalyst was essential for the synthesis of anthracene derivatives, as the template reaction was unsuccessful without nickel or cobalt. Under optimized conditions, a variety of substrates featuring functional groups such as alkyl and alkene chains, hydroxy groups, and benzene and pyridine rings were examined, resulting in the successful synthesis of the desired anthracene products in good to high yields (Scheme 17).

**SCHEME 18:**
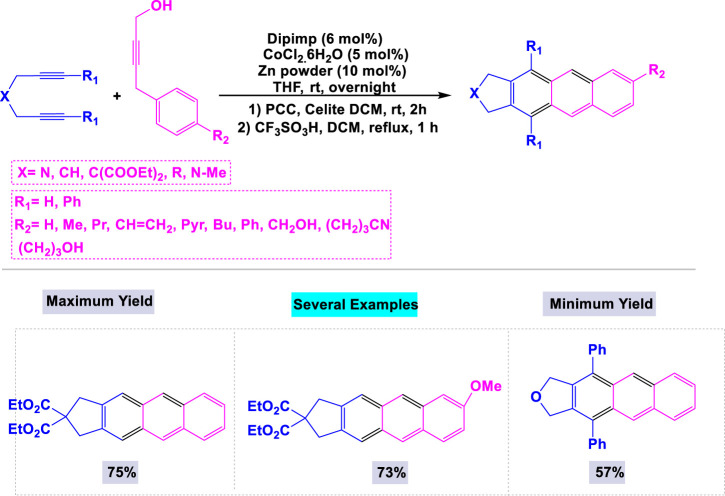
Synthesis of substituted anthracenes through oCl_2_·6H_2_O/Zn reagent catalyzed [2 + 2 + 2] cycloaddition reaction of 1,6-diynes with 4-aryl-2-butyn-1-ols.

In a separate publication, Saino et al. (2010) reported that the combination of CoCl_2_·6H_2_O and Zn powder constitutes an efficient system for synthesizing substituted anthracenes through [2 + 2 + 2] alkyne-cyclotrimerization reactions with 2-iminomethylpyridine (dipimp) (Hoffmann et al., 2019). When the reaction was conducted using only the cobalt catalyst, the yield of the target product was unsatisfactory. As illustrated in Scheme 18, the [2 + 2 + 2] cycloaddition reaction of 1,6-diynes with 4-aryl-2-butyn-1-ols, catalyzed by the CoCl_2_·6H_2_O/Zn reagent in the presence of dipimp, resulted in the formation of the desired substituted anthracenes with good yields. Notably, this catalytic system exhibited high activity in the synthesis of substituted pentaphenes and trinaphthylenes.

**SCHEME 19:**
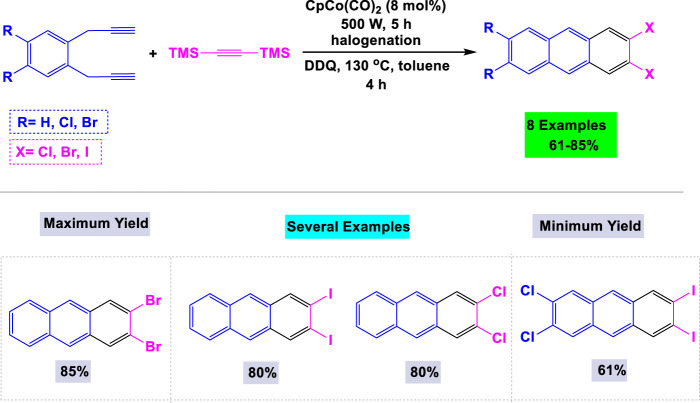
Synthesis of 2,3- and 2,3,6,7-halogenated anthracenes through cobalt-catalyzed [2 + 2 + 2] cyclotrimerization reactions with bis(trimethylsilyl)acetylenes.

In 2019, Hoffmann and colleagues established an effective synthetic route for the preparation of 2,3- and 2,3,6,7-halogenated anthracenes through cobalt-catalyzed [2 + 2 + 2] cyclotrimerization reactions employing bis(trimethylsilyl)acetylenes (Koley et al., 2023). As depicted in Scheme 19, a crucial step involved the introduction of chlorine, bromine, or iodine substituents via halodesilylation of TMS-substituted cyclotrimerization adducts. The synthesis of 2,3- and 2,3,6,7-halogenated anthracenes achieved satisfactory yields through oxidation and aromatization processes using DDQ. Notably, this method exhibited high efficiency in producing 2,3,6,7-halogenated anthracene derivatives, which are typically difficult to obtain.

### 4.5 Gold catalyzed synthesis of anthracenes

**SCHEME 20:**
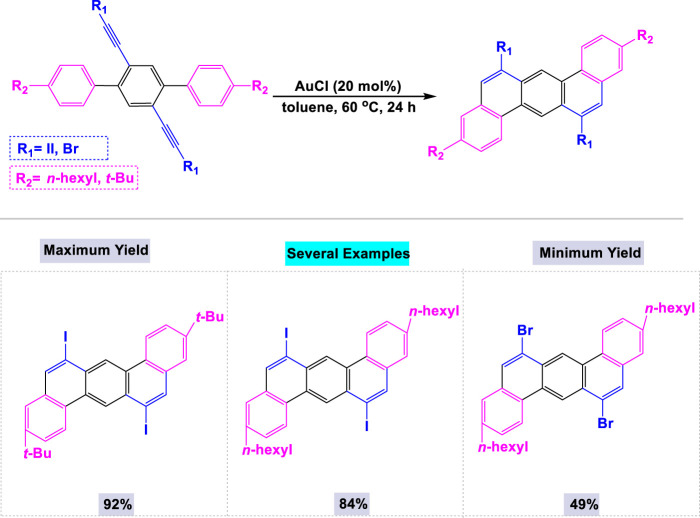
Synthesis of dibenzo [a, h]anthracenes through one-pot double cyclization reactions in the presence of catalytic amount of AuCl.

Gold-catalyzed synthesis is a specialized area of organic chemistry that utilizes gold as a catalyst to facilitate a range of chemical transformations, particularly the activation of alkynes, allenes, and alkenes (Nakae et al., 2012). This methodology is highly regarded for its capacity to promote mild and selective reactions, enabling the efficient synthesis of complex organic molecules with potential applications in drug development and materials science. In 2012, Nakae and colleagues reported the synthesis of a series of dibenzo[a, h]anthracenes through one-pot double cyclization reactions employing a catalytic amount of AuCl (Scheme 20). (Shu et al., 2013) The presence of gold catalysts was essential for these reactions, as the template double cyclization of dihaloethynylterphenyl could not be achieved in the absence of gold. These one-pot double cyclization reactions were performed using 20 mol% of AuCl in toluene at 60°C for 24 h.

**SCHEME 21:**
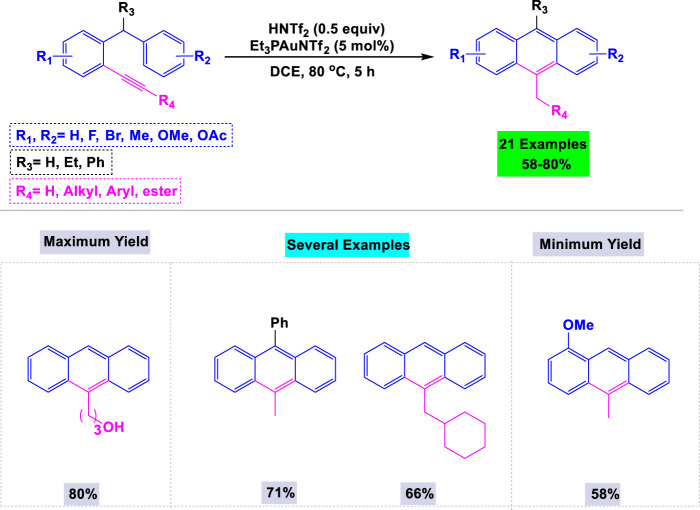
Synthesis of substituted anthracenes through gold catalyzed cyclization of o-alkynyldiarylmethanes.

Shu and colleagues have developed a novel synthetic procedure for preparing substituted anthracenes via the cyclization of o-alkynyldiarylmethanes using a catalytic amount of the gold complex (Et_3_PAuNTf_2_) (Sawano et al., 2019). They conducted template cyclization of o-alkynyldiarylmethane under various conditions to identify the optimal parameters for synthesizing substituted anthracenes. Notably, the template product was not observed in the absence of gold catalysts. Under the standardized conditions outlined in Scheme 21, functionalities such as F, Br, and Me, as well as the acid-sensitive OAc group on the aromatic ring, were all well tolerated during the cyclization process.

### 4.6 Iridium catalyzed synthesis of anthracene derivatives

**SCHEME 22:**
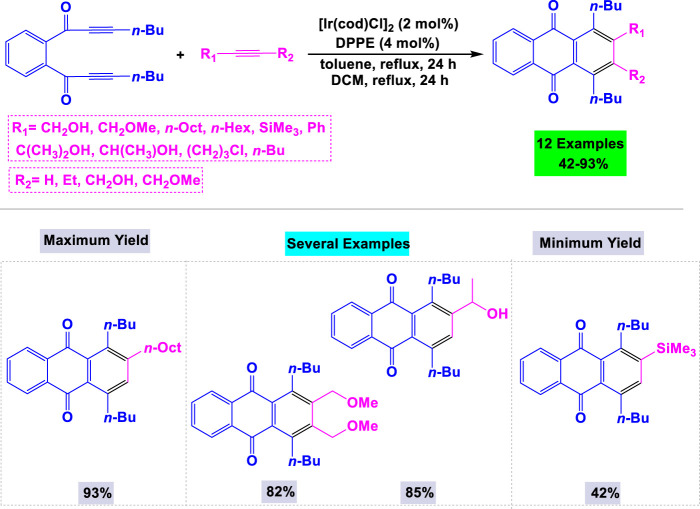
Synthesis of anthraquinone derivatives through [Ir (cod)Cl]_2_/DPPE catalyzed [2 + 2 + 2] cycloaddition of a 1,2-bis(propiolyl)benzene derivative with terminal and internal alkynes.

Takeuchi and colleagues have developed a valuable synthetic method for producing biologically promising anthraquinone derivatives (Fukutani et al., 2009). This approach utilizes [Ir (cod)Cl]_2_ (where cod is 1,5-cyclooctadiene) in combination with bis(diphenylphosphino)ethane (DPPE) as an effective catalytic system. The reaction of a 1,2-bis(propiolyl)benzene derivative with three equivalents of 1-hexyne was explored under various conditions, revealing that the absence of ligands resulted in a poor yield of the target product. As illustrated in Scheme 22, a range of anthraquinone derivatives can be synthesized in moderate to high yields through the [Ir (cod)Cl]_2_/DPPE-catalyzed [2 + 2 + 2] cycloaddition of a 1,2-bis(propiolyl)benzene derivative with terminal and internal alkynes in nonpolar solvents under reflux conditions.

### 4.7 Rhodium catalyzed synthesis of anthracenes

**SCHEME 23:**
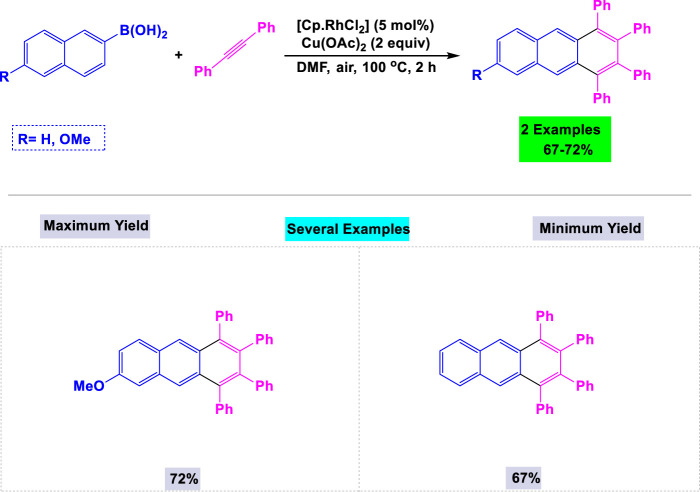
Synthesis of 1,2,3,4-tetrasubtituted anthracene derivatives through rhodium-catalyzed oxidative coupling reactions of aryl-boronic acids with internal alkynes in the presence of Cu(OAc)_2_ as the oxidant.

In 2009, Fukutani and his team developed a general and efficient method for synthesizing 1,2,3,4-tetrasubstituted anthracene derivatives through rhodium-catalyzed oxidative coupling reactions between aryl boronic acids and internal alkynes (Scheme 23). (Zhang et al., 2016) They investigated the effects of various catalysts and solvents to optimize the reaction conditions. This catalytic system also demonstrated high activity in the synthesis of other polysubstituted fused aromatic compounds.

**SCHEME 24:**
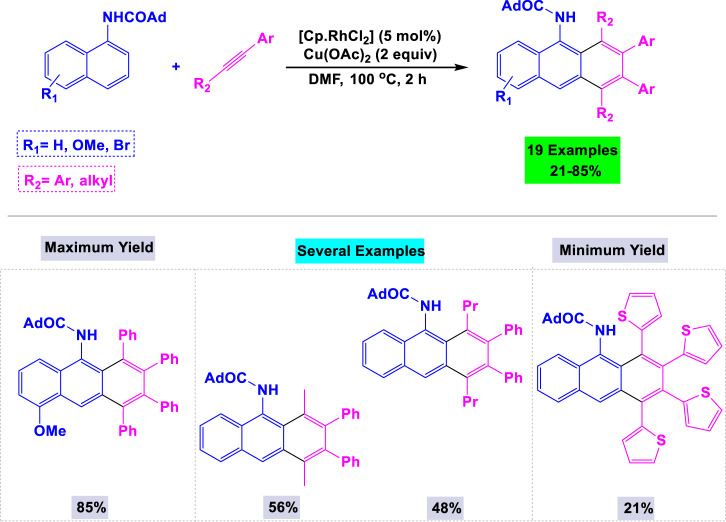
Synthesis of substituted anthracenes through rhodium-catalyzed oxidative benzannulation reactions of 1-adamantoyl-1-naphthylamines with internal alkynes in the presence of Cu(OAc)_2_ as the oxidant.

Zhang and colleagues developed an innovative and highly efficient synthetic methodology for the preparation of substituted anthracenes through rhodium-catalyzed oxidative benzannulation reactions. This process involves the reaction of 1-adamantoyl-1-naphthylamines with internal alkynes in the presence of Cu(OAc)_2_ as the oxidant, utilizing DMF under thermal conditions (Scheme 24) (Kitazawa et al., 2011). The reactions proceeded smoothly with alkynes containing either electron-donating or electron-withdrawing groups on the benzene ring, resulting in the formation of target anthracene products in moderate to good yields.

### 4.8 Ruthenium catalyzed synthesis of anthracenes

**SCHEME 25:**
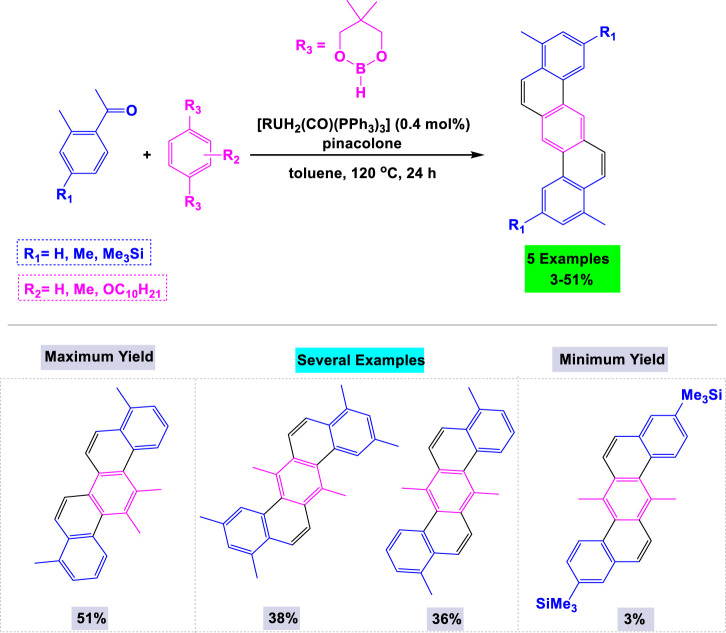
Synthesis of dibenzo [*a,h*] anthracenes through [RuH_2_(CO) (PPh_3_)] catalyzed one-pot regioselective C¬H arylation of aromatic ketones.

In 2011, Kitazawa and colleagues reported a one-pot, regioselective C–H arylation of aromatic ketones utilizing [RuH_2_(CO) (PPh_3_)] as the catalyst for the synthesis of dibenzo [*a*,*h*]anthracenes ^159^ This reaction was performed in pinacolone under reflux conditions. Additionally, they explored the effects of various substituents at specific positions on arenediboronates under standardized conditions (Scheme 25).

## 5 Conclusion

The synthesis of anthracene scaffolds catalyzed by transition metals has witnessed significant advancements in recent years, enhancing the efficiency, selectivity, and sustainability of these important organic compounds. Transition metal catalysis has emerged as a powerful tool for developing innovative methodologies that address challenges associated with traditional synthetic routes.

Recent achievements in this field include the implementation of C–H activation strategies, which allow for direct functionalization of anthracene derivatives, simplifying synthetic pathways and reducing the need for pre-functionalization. The evolution of cross-coupling reactions, especially using palladium and nickel catalysts, has enabled the efficient construction of complex anthracene-based structures with high yields and selectivity.

New catalytic systems, including earth-abundant metals and chiral catalysts, have further expanded the scope of anthracene synthesis, providing access to enantiomerically enriched compounds valuable in pharmaceuticals and materials science. The integration of green chemistry principles into these methodologies has led to sustainable practices, minimizing waste and environmental impact.

Looking forward, the future of anthracene scaffold synthesis appears promising, with ongoing efforts to optimize methodologies, develop novel catalysts, and explore new reaction conditions. These advancements not only enhance the utility of anthracene derivatives in various applications but also contribute to broader goals of sustainable and efficient organic synthesis. Collaboration between chemists, materials scientists, and industry will drive further innovations in this dynamic field, paving the way for new discoveries and applications of anthracene-based compounds.

Recent achievements underscore the dynamic nature of this field and its critical role in advancing modern organic synthesis. The continued exploration and optimization of these catalytic processes will pave the way for new discoveries and applications, reinforcing the importance of transition metals in synthesizing complex molecular architectures.
